# Electrochemical Energy Storage Application and Degradation Analysis of Carbon-Coated Hierarchical NiCo_2_S_4_ Core-Shell Nanowire Arrays Grown Directly on Graphene/Nickel Foam

**DOI:** 10.1038/srep20264

**Published:** 2016-02-01

**Authors:** Rujia Zou, Muk Fung Yuen, Li Yu, Junqing Hu, Chun-Sing Lee, Wenjun Zhang

**Affiliations:** 1 State Key Laboratory for Modification of Chemical Fibers and Polymer Materials, College of Materials Science and Engineering, Donghua University, Shanghai 201620, China; 2 Center of Super-Diamond and Advanced Films (COSDAF), Department of Physics and Materials Science, City University of Hong Kong, Hong Kong

## Abstract

We developed a new electrode comprising thin carbon layer coated hierarchical NiCo_2_S_4_ core-shell nanowire arrays (NiCo_2_S_4_@C CSNAs) on graphene/Ni foam (Ni@G) substrates. The electrode showed outstanding electrochemical characteristics including a high specific capacitance of 253 mAh g^−1^ at 3 A g^−1^, high rate capability of 163 mAh g^−1^ at 50 A g^−1^ (~64.4% of that at 3 A g^−1^), and long-term cycling stability with a capacity retention of 93.9% after 5000 cycles. Comparative studies on the degradation of hierarchical NiCo_2_S_4_ CSNA electrodes with and without carbon coatings revealed that the morphology pulverization, structural separation at core/shell interface, and irretrievably chemical composition change of NiCo_2_S_4_ CSNAs electrode are major factors that deteriorate the electrochemical performance of the electrodes without carbon coating. The favorable roles of carbon coatings on hierarchical NiCo_2_S_4_ CSNAs were further clarified: (1) serving as a physical buffering layer that suppresses the structural breakdown; (2) retarding the chemical composition conversion of the NiCo_2_S_4_ CSNAs; and (3) providing extra path for charge transition in addition to the NiCo_2_S_4_ core nanowires. Understanding of the degradation mechanisms and the significance of the surface carbon coatings would provide useful guidelines for the design of new electrode materials for high-performance electrochemical devices.

With the rapid development of portable devices, green energy harvesting, and electric automobiles, safe energy storage devices with improved power and energy densities are becoming increasingly important. Electrochemical devices are very promising energy storage devices, due to their short charging time, high power densities, and long lifespans^1^,^2^. Among all these power sources, nanostructured electrode batteries and supercapacitors are the paradigm devices as they are promising to deliver power source with both high energy and power densities. The increasing applications of electrochemical devices in electronic devices and industries require on further improvement of the energy density, rate capability, safety, and durability of electrodes, which to a large extent rely on the development of new electrode materials and novel electrode structures. Transition metal oxides (MOs such as RuO_2_, MnO_2_, NiO, CoO) and sulfides (MSs such as CoS, NiS, WS_2_ etc.) from an important group of electrode materials due to their abundant reversible redox reactions, high theoretical capacity, and long cycle life^3^,^4^,^5^,^6^,^7^,^8^,^9^,^10^,^11^,^12^. Recently, the ternary cobalt nickel sulfides (NiCo_2_S_4_) has drawn great research interest because of their intriguing advantages over the binary MOs and MSs^2^,^13^,^14^. For instance, NiCo_2_S_4_ have an electrical conductivity of at least four orders of magnitude higher than that of either nickel oxides/sulfides or cobalt oxides/sulfides. High electrical conductivity could decrease the charge transfer resistance of the electrodes, and thus leading to increased power density^15^,^16^. In addition, with the contributions from both nickel and cobalt ions, NiCo_2_S_4_ are expected to offer richer redox reactions than the binary sulfides and oxides and thus increased capacity.

Various NiCo_2_S_4_ nanostructures, including nanoparticles^17^,^18^, nanosheets^19^,^20^, nanotubes^21^, and microspheres^22^, have been synthesized. A common approach to apply these NiCo_2_S_4_ nanostructures as electrodes in electrochemical devices is to mix them with polymeric binder and carbon black and press the mixture onto current collectors. However, the use of conductive additive and binder inevitably increases the undesirable interparticle resistance which decreases the efficiency of electron transport among active materials^23^,^24^. Recently, it was shown that direct growth of NiCo_2_S_4_ nanowire arrays on metallic substrates could overcome the drawbacks from mixing active materials with conductive additive and binder, and led to improved capacity and cyclic stability^25^,^26^. Though NiCo_2_S_4_ is important electrode material from those reported^17^,^18^,^19^,^20^,^21^,^22^, so far the change of electrochemical reactions on the surface/inside of NiCo_2_S_4_ materials has not been studied systematically. Compared with one dimensional (1D) nanostructure, three dimensional (3D) heterostructure will be able to more fully be present in the change of electrode materials during electrochemical reactions^27^,^28^,^29^. In this work, we synthesized for the first time a thin carbon layer coated three dimensional (3D) hierarchical NiCo_2_S_4_ core-shell nanowire arrays (NiCo_2_S_4_@C CSNAs) directly on graphene-coated Ni (Ni@G) foam. In comparison with the NiCo_2_S_4_ nanowire arrays on metallic substrates, the effective surface area of the active electrode materials was greatly increased, and thus the electrochemical performances of the electrode was significantly improved, e.g., a high specific capacitance of 253 mAh g^−1^ at 3 A g^−1^, high rate capability of 163 mAh g^−1^ at 50 A g^−1^ (~64.4% of that at 3 A g^−1^), and long-term cycling stability with a capacity retention of 93.9% after 5000 cycles. We further studied comparatively the degradation mechanisms of hierarchical NiCo_2_S_4_ CSNAs electrodes with and without carbon coatings, and revealed favorable roles of carbon coatings in maintaining the structural integrity and chemical composition of the hierarchical NiCo_2_S_4_ CSNAs during charge/discharge cycles.

## Results

**Figure 1:**
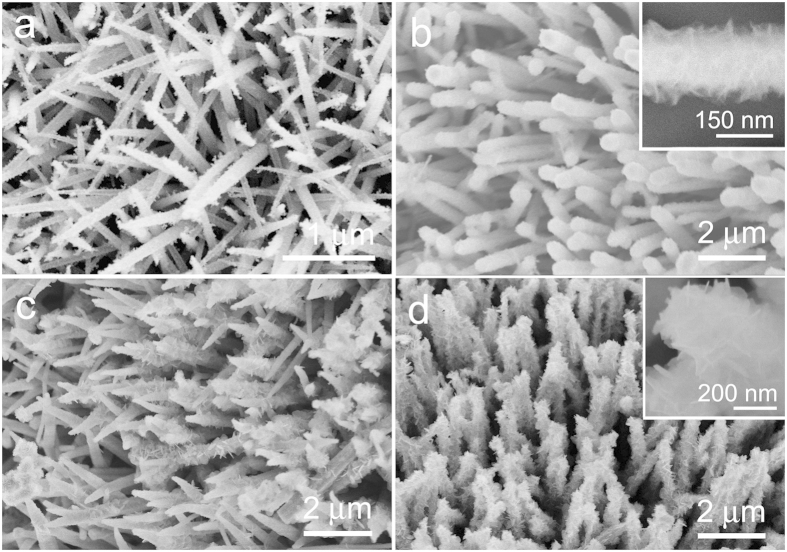
SEM images of the nanowire arrays obtained for different sulfurization time: **(a)** 12 h, **(b)** 24 h, **(c)** 36 h and **(d)** 48 h. Insets in **(b**,**d)** show the magnified images of hierarchical NiCo_2_S_4_ CSNAs after sulfurization for 24 and 48 h, respectively.

The hierarchical NiCo_2_S_4_ CSNAs were synthesized by hydrothermal method followed by sulfurization treatment (see the Supplementary Information I for Experimental details). Ni@G foams were used as substrates (Figure S1), and graphene was pre-coated on Ni foam to protect it which would otherwise become fragile during the sulfurization. For the synthesis of hierarchical NiCo_2_S_4_ CSNAs, the Ni@G foam was first immersed into an aqueous solution containing Ni^2+^, Co^2+^ and urea, and the (Ni, Co) hydroxide nanowire arrays were grown uniformly on the surface of Ni@G foam, as shown in Figure S2. Then, the (Ni, Co) hydroxide precursor nanowire arrays were converted to highly-ordered hierarchical NiCo_2_S_4_ CSNAs via a facile sulfurization. Finally, the samples were annealed at 320 ^o^C in Ar gas to get crystallized NiCo_2_S_4_ CSNAs. As shown in Fig. 1a, the NiCo_2_S_4_ nanowires on Ni@G foam possessed an average diameter of ~70 nm and length up to ~6 μm, and their surfaces were relatively smooth after sulfurization for 12 h. After sulfurization for 24 h, the NiCo_2_S_4_ nanowires still remained the array structure, but the surface of nanowires became rough, as shown in Fig. 1b. Enlarged SEM image (inset in Fig. 1b) reveals that the nanowire surface was uniformly covered by clumps (a mat) of NiCo_2_S_4_ nanosheets (as confirmed by XRD and HRTEM observations below), forming a core-shell hierarchical nanostructure. As the sulfurization was prolonged to 36 h, the size of NiCo_2_S_4_ nanosheets increased. However, not all NiCo_2_S_4_ nanowires were covered by nanosheets. The uniform and large-scale NiCo_2_S_4_ CSNAs on Ni@G foam were obtained after sulfurization for 48 h, as shown in Fig. 1d. Enlarged SEM image inset Fig. 1d reveals that ultrathin NiCo_2_S_4_ nanosheets have a length of ~200 nm and a thickness of ~8 nm.

**Figure 2:**
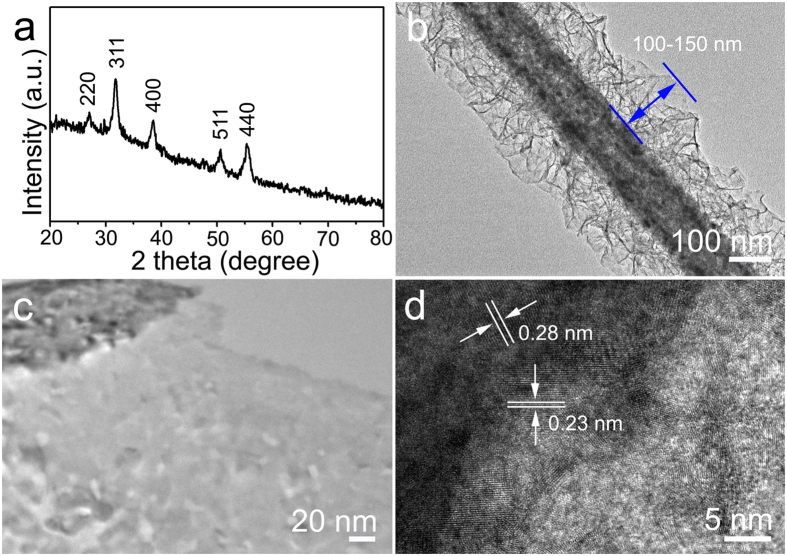
Characterization of the hierarchical NiCo_2_S_4_ core-shell nanowires. (**a**) XRD pattern of the hierarchical NiCo_2_S_4_ core-shell nanowires scratched from Ni@G foam. **(b)** TEM image of the hierarchical NiCo_2_S_4_ core-shell nanowire, **(c)** Enlarged TEM view and **(d)** HRTEM image of the joint area between NiCo_2_S_4_ nanowire and nanosheet.

Figure 2a shows XRD pattern of the as-synthesized hierarchical NiCo_2_S_4_ CSNAs scratched from Ni@G foam after sulfurization for 48 h. The diffraction peaks at 26.8°, 31.6°, 38.3°, 50.5° and 55.3° were indexed to the 220, 311, 400, 511, and 440 planes of the cubic NiCo_2_S_4_ with reference to the standard diffraction pattern of NiCo_2_S_4_ (Joint Committee on Powder Diffraction Standards (JCPDS) No. 20–0782). No other diffraction peaks were observed, verifying the formation of phase-pure NiCo_2_S_4_. The morphology and structure of hierarchical NiCo_2_S_4_ CSNAs after sulfurization for 48 h were further investigated by using TEM. Consistent with the SEM observations above, the low-magnification TEM images (Fig. 2b and Figure S3) confirm the formation of uniform core-shell hierarchical structure with the NiCo_2_S_4_ hollowed nanowire as core and NiCo_2_S_4_ nanosheets (height up to ~100–150 nm) as shell. Interestingly, it is noted that all of the NiCo_2_S_4_ core nanowires are mesoporous structure which is resulted from the replacement of O^2−^ by S^2−^ through sulfurization process^30^. The mesopores with a size ranging from 2 to 8 nm are uniformly distributed in the nanowires and nanosheets (Fig 2b,c), implying that the sulfurization processes homogeneously in the bulk of nanowires. HRTEM image in Fig. 2d further verified the crystal characteristics of NiCo_2_S_4_ nanostructures. As denoted in this figure, the interplane distances were measured to be 0.28 nm and 0.23 nm, which correspond to the *d*-spacing of (311) and (400) planes of cubic NiCo_2_S_4_, respectively. The TEM observations verified that the hierarchical nanostructure is composed of the same electrochemical materials in core and shell, wherein both the nanowire core and the nanosheets shell could contribute to electrochemical reactions for energy storage. Moreover, the hierarchical NiCo_2_S_4_ core-shell nanowires are featured with open spaces between shell nanosheets, which allow access of electrolyte to both core and shell surfaces and leads to improved electrochemical performance of active materials.

The surface area and porosity of hierarchical NiCo_2_S_4_ CSNAs scratched from Ni@G foam were examined with adsorption-desorption experiment using Brunauer-Emmett-Teller (BET) method (Figure S4). Following the Barrett-Joyner-Halenda (BJH) method, NiCo_2_S_4_ core-shell nanowires exhibited a pore size distribution ranging from 4 to 8 nm, which is consistent with the TEM observation in Fig. 2c. The specific surface area of hierarchical NiCo_2_S_4_ core-shell nanowires was calculated to be 84.9 m^2^/g, which is significantly higher than those of the other NiCo_2_S_4_ nanostructures reported in literatures^18^,^19^,^31^,^32^. A high specific surface area of active materials enables the efficient contact with electrolyte and increases the numbers of electroactive for charge storage and delivery.

The performance of electrochemical electrodes is known to depend strongly on the specific surface area and a well the electrical conductivity of the active materials^1^,^2^. In this work, the electric conductivity of an individual hierarchical NiCo_2_S_4_ core-shell nanowire was measured *in situ* in TEM using a specially design holder (Nanofactory Instruments AB). The experimental setup is sketched in Figure S5a, and the other details have been reported in our previous work^33^,^34^. The hierarchical NiCo_2_S_4_ core-shell nanowire bridged Au tip and Pt probe by precisely adjusting Pt probe using a piezo-driven manipulator, as shown in Figure S5b. For comparison, a NiCo_2_O_4_ nanowire without nanosheets shell was also measured using the same method (inset Figure S5b, see the Supplementary Information II for Experimental details). The corresponding *I-V* curves were shown in Figure S5c. Both of the voltage range from −1.0 to 1.0 V, the current value of hierarchical NiCo_2_S_4_ core-shell nanowire varies from ~ −97.2 to 84.4 μA, while the current of NiCo_2_O_4_ nanowire varies from ~ −0.39 to 0.35 μA. Thus, the electrical conductivity of the NiCo_2_S_4_ core-shell nanowire is over 300 times higher than that of NiCo_2_O_4_ nanowire, and about 4–5 orders of magnitude higher than those of metal oxides and sulfides in our previous work^35^,^36^.

**Figure 3:**
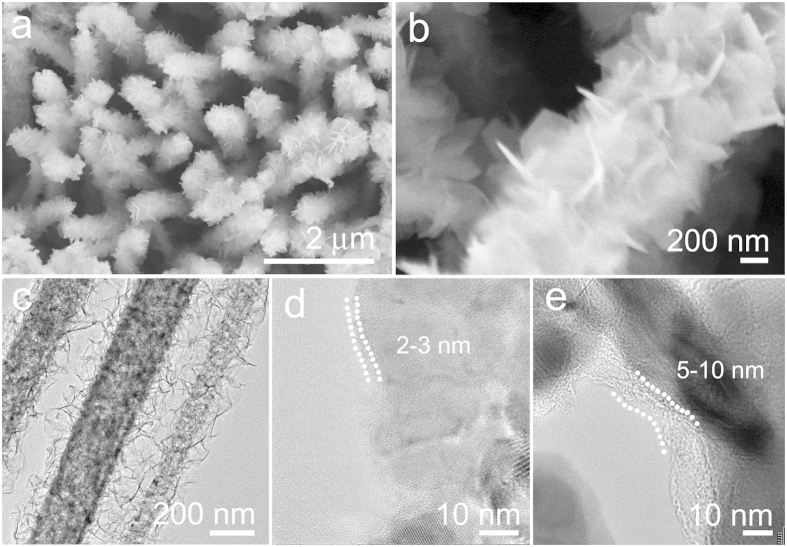
Characterization of the hierarchical NiCo_2_S_4_@C CSNAs. (**a**,**b)** SEM images of the hierarchical NiCo_2_S_4_@C CSNAs after carbon coating by hydrothermal reaction for 3h. **(c)** TEM image of the carbon-coated hierarchical NiCo_2_S_4_@C CSNAs after hydrothermal deposition for 3h. HRTEM images of hierarchical NiCo_2_S_4_@C CSNAs samples with carbon layers deposited by hydrothermal reaction for: **(d)** 3 h and **(e)** 8 h.

A layer of carbon shell was deposited onto the surface of hierarchical NiCo_2_S_4_ CSNAs by hydrothermal method. As shown in Fig. 3a, the hierarchical NiCo_2_S_4_ CSNAs retained their morphology well after reaction for 3 h (see the Supplementary Information I for Experimental details). Enlarged SEM image in Fig. 3b reveals that the thickness of NiCo_2_S_4_ nanosheets is increased to 10–12 nm after carbon coating. Agreeing with the SEM observations, the low-magnification TEM image in Fig. 3c also demonstrates that the NiCo_2_S_4_ nanowires still maintain the core-shell structure with the nanowire cores uniformly covered by NiCo_2_S_4_. As further verified by the HRTEM image in Fig. 3d, a thin carbon layer shell with a thickness of 2–3 nm was deposited over the surface of hierarchical NiCo_2_S_4_ CSNAs. The thickness of carbon layer could be controlled by reaction time, and it increased to 4–8 nm after reaction for 8 h (Fig. 3e).

**Figure 4:**
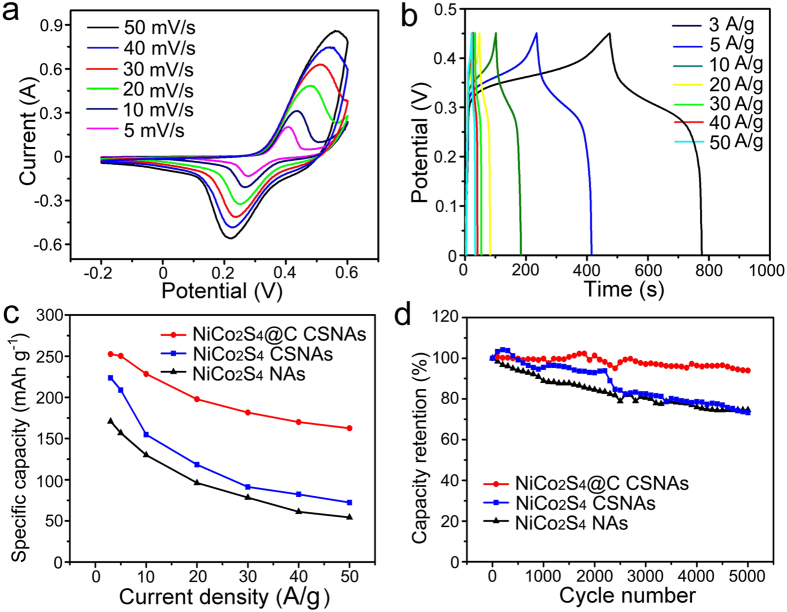
Electrochemical properties of NiCo_2_S_4_@C CSNAs, NiCo_2_S_4_ CSNAs and NiCo_2_S_4_ NAs electrodes in 1 M KOH aqueous solution at room temperature. (**a**) The CV curves of NiCo_2_S_4_@C CSNAs electrode at different scan rates. (**b**) The CD curves of NiCo_2_S_4_@C CSNAs electrode at different current densities. (**c**) Specific capacities of NiCo_2_S_4_@C CSNAs (red curve), NiCo_2_S_4_ CSNAs (blue curve) and NiCo_2_S_4_ NAs (black curve) electrodes at different current densities. (**d**) Cycling properties of NiCo_2_S_4_@C CSNAs (red curve), NiCo_2_S_4_ CSNAs (blue curve) and NiCo_2_S_4_ NAs (black curve) electrodes at a scan rate of 50 mVs^−1^ in 5000 cycles.

The electrochemical performance of hierarchical NiCo_2_S_4_@C CSNAs on Ni@G foam was studied in a standard three-electrode configuration using 1.0 M KOH electrolyte. The control experiments on reference samples of hierarchical NiCo_2_S_4_ CSNAs and NiCo_2_S_4_ nanowire arrays (NAs) on Ni@G foams were also performed (see the Supplementary Information III for Experimental details and Figure S6). Because the 4–8 nm thick carbon layer restrain the ions in electrolyte to penetrate through it and react with the NiCo_2_S_4_ CSNAs and leads to poor electrochemical performance (Figure S7), the following tests were conducted on the hierarchical NiCo_2_S_4_@C CSNAs with a thin carbon layer of 2–3 nm. Figure 4a presents the cyclic voltammetry (CV) curves of hierarchical NiCo_2_S_4_@C CSNAs electrode at different scan rates of 5, 10, 20, 30, 40 and 50 mV s^−1^ in a potential window of −0.2–0.6 V. The redox current increased with the increase of scan rate from 5 to 50 mV s^−1^, and the oxidation and reduction peaks shifted toward higher and lower potentials, respectively, resulting in an enlarged potential separation. The CV curves of hierarchical NiCo_2_S_4_ CSNAs and NiCo_2_S_4_ NAs electrodes displayed the same tendency at different scan rates (Figure S8a), which suggests the same redox mechanism as that of hierarchical NiCo_2_S_4_@C CSNAs. However, at the same scan rate, the NiCo_2_S_4_@C CSNAs electrode has obviously a much larger integrated area than those of NiCo_2_S_4_ CSNAs and NiCo_2_S_4_ NAs electrodes, which indicates that the hierarchical NiCo_2_S_4_@C CSNAs electrode has a higher capacity. We also studied the CV characteristics of pure Ni@G foam, as shown in Figure S9. It was revealed that the pure Ni@G foam exhibited a much low capacity, indicating that the Ni@G foam had negligible contribution to the overall capacity of hierarchical NiCo_2_S_4_@C CSNAs electrode.

The galvanostatic charge-discharge (CD) tests of NiCo_2_S_4_@C CSNAs, NiCo_2_S_4_ CSNAs and NiCo_2_S_4_ NAs were performed in a potential window between 0 and 0.45 V at current densities ranging from 3 to 50 A g^−1^, as shown in Fig. 4b and Fig. S8. While close observation of the CV and CD curves of NiCo_2_S_4_@C CSNAs and NiCo_2_S_4_ CSNAs electrodes suggested both capacitive effect and ion-diffusion effect for the energy storage, further analysis on the dependence of the current (*i*) with the sweep rate (*v*) at a fixed potential in reduction processes verified that they are closer to the battery-type materials (Figure S10)^37^,^38^,^39^. So, in order to obtain real and accurate values of the energy storage, the capacities were calculated based on the battery equation (unit, mAh g^−1^). According to previously reported^40^, the specific capacity values of the electrode materials are calculated from the galvanostatic discharge curves at various current densities. The specific capacity of NiCo_2_S_4_@C CSNAs was calculated to be 253, 250, 228, 198, 182, 170, and 163 mAh g^−1^ at current densities of 3, 5, 10, 20, 30, 40, and 50 A g^−1^, respectively, as shown in Fig. 3c (red curve). The specific capacity of NiCo_2_S_4_ CSNAs and NiCo_2_S_4_ NAs electrodes at different current densities were also evaluated, as depicted by blue and black curves, respectively, in Fig. 4c. The specific capacity of hierarchical NiCo_2_S_4_@C CSNAs electrode is apparently higher than those of NiCo_2_S_4_ CSNAs and NiCo_2_S_4_ NAs electrodes for each current density. As the current densities increased from 3 to 50 A g^−1^, the rate capability of NiCo_2_S_4_@C CSNAs, NiCo_2_S_4_ CSNAs and NiCo_2_S_4_ NAs electrodes were reduced to 64.4%, 32.3% and 31.7%, respectively, of their original values, indicating that NiCo_2_S_4_@C CSNAs electrode has best rate capability. As a result, at the current density of 50 A g^−1^, the specific capacity of the NiCo_2_S_4_@C CSNAs ~2.3 times higher than that of the is NiCo_2_S_4_ CSNAs (163 mAh g^−1^ vs. 72 mAh g^−1^). The cycling stability of NiCo_2_S_4_@C CSNAs, NiCo_2_S_4_ CSNAs and NiCo_2_S_4_ NAs electrodes were evaluated by repeated charge/discharge measurements at a scan rate of 50 mVs^−1^, as shown in Fig. 4d. Interestingly, the overall loss of capacity of NiCo_2_S_4_@C CSNAs after 5000 cycles was less than 6.1%, i.e., the capacity retention of 93.9% over 5000 cycles, which is obviously superior to the NiCo_2_S_4_ CSNAs (73.3%) and NiCo_2_S_4_ NAs (74.6%) electrodes. The capacity retention of NiCo_2_S_4_ NAs is slightly higher than that of hierarchical NiCo_2_S_4_ CSNAs. On the one hand, high specific surface areas of active materials of NiCo_2_S_4_ CSNAs enable the efficient contact with electrolyte, and increase the electroactive sites for redox reactions. On the other hand, compared with NiCo_2_S_4_ NAs, hierarchical NiCo_2_S_4_ CSNAs may result in structural change of nanosheets from nanowire cores more easily.

## Discussion

The above results revealed that the hierarchical NiCo_2_S_4_ CSNAs electrodes have the following characteristics which have been demonstrated to be beneficial to improve the electrochemical perfromance^27^,^28^,^29^,^41^,^42^,^43^,^44^,^45^. First, both core and shell are active materials; and the 3D core-shell structure enables easy access of electrolyte. Therefore, both of them can effectively contribute to the capacity. Secondly, the hierarchical NiCo_2_S_4_ CSNAs are highly conductive, which can provide “superhighways” for charge in the core-shell structure. The direct growth of NiCo_2_S_4_ nanoshells on NiCo_2_S_4_ nanowires avoids the use of polymer binder/conductive additives and further guarantees the effective charge transport between them. Moreover, the NiCo_2_S_4_ shells and NiCo_2_S_4_ nanowires are mesoporous that increases the electroactive sites. Therefore, the hierarchical NiCo_2_S_4_ CSNAs/Ni@G electrode has a comparable or even superior performance to the previously reported electrodes based on nickel/cobalt sulfide nanomaterials^40^. Surface carbon coating could significantly further improve the overall electrochemical properties, and made the hierarchical NiCo_2_S_4_@C CSNAs/Ni@G a very promising candidate for electrode applications.

**Figure 5:**
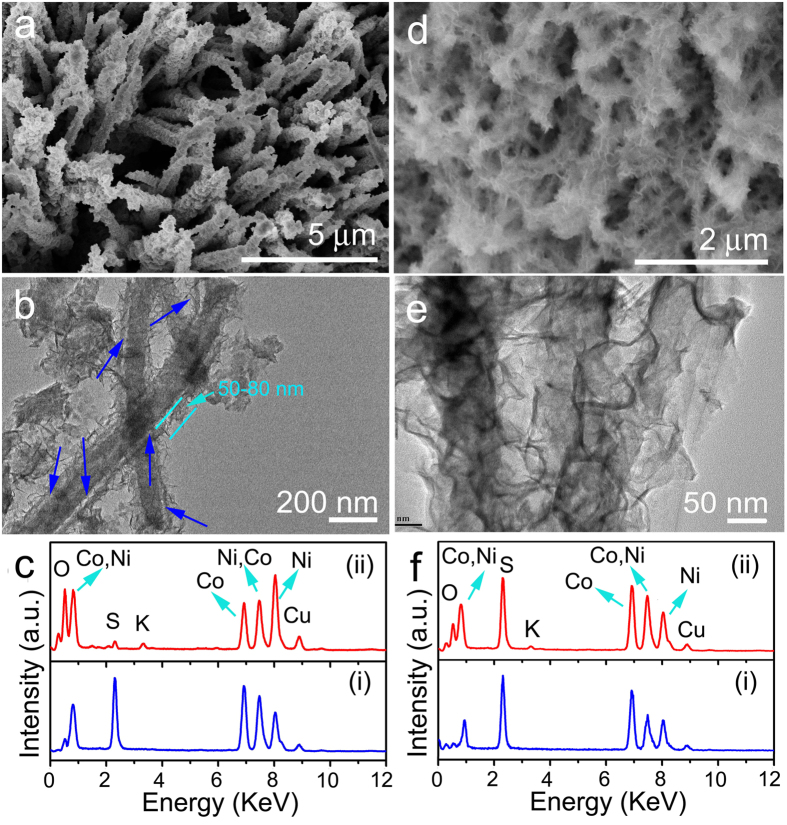
Characterization of the hierarchical NiCo_2_S_4_ CSNAs and NiCo_2_S_4_@C CSNAs after charge/discharge for 5000 cycles at a scan rate of 50 mV s^−1^. (**a**) SEM image, (**b**) TEM image, and (**c**) EDS pattern of the NiCo_2_S_4_ CSNAs electrode after charge/discharge for 5000 cycles at a scan rate of 50 mV s^−1^. (**d**) SEM image, (**e**) TEM image, and (**f**) EDS pattern of the NiCo_2_S_4_@C CSNAs electrode after charge/discharge for 5000 cycles at a scan rate of 50 mV s^−1^.

In order to understand the degradation of electrochemical performance and beneficial role of surface carbon coating, we comparatively analyzed the variation of microstructure and chemical composition of the hierarchical NiCo_2_S_4_ CSNAs with and without carbon coating after 5000 charge/discharge cycles. The structures of NiCo_2_S_4_ CSNAs and NiCo_2_S_4_@C CSNAs electrodes after cycling at 50 mVs^−1^ were characterized by SEM and TEM. The morphology of the NiCo_2_S_4_ CSNAs mainly retained their array structure, but the increased diameter and roughness of NiCo_2_S_4_ nanowires after 5000 cycles, as shown in Fig. 5a and enlarged SEM image in Figure S11. However, TEM observation in Fig. 5b (more NiCo_2_S_4_ core-shell nanowires in Figure S12) reveals that the NiCo_2_S_4_ nanosheets were shortened to ~50–80 nm in height, and shell nanosheets were detached from the core nanowires, forming a nanometer-scale gap between core and shell after 5000 cycles gaps, as denoted by the blue arrows. The breakdown of the hierarchical core-shell structure could possibly be caused by the repeated charge transfer through the core-shell interface during cycling processes which might induce uneven Joule heating and anisotropic volume expansion of nanowires and nanosheets of different geometric features.

The redox reactions during repeated charge/discharge processes may lead to dissolution of NiCo_2_S_4_ into the electrolyte, and an irretrievably chemical composition change of the NiCo_2_S_4_ . As shown by the EDS spectra for NiCo_2_S_4_ CSNAs electrode before (i) and after (ii) 5000 cycles in Fig. 5c, the contents of Ni and S decreased, and O content increased drastically after cycling, suggesting the a variation of the chemical composition of NiCo_2_S_4_ (Figure S13). Figure S14 shows representative CV curves of NiCo_2_S_4_ CSNAs electrode in the voltage window of −0.2 to 0.6 V at a scan rate of 50 mV s^−1^ for the 1^st^, 1000^th^, 2000^th^, 3000^th^, 4000^th^ and 5000^th^ cycles. The locations of the pair of current peaks for faradic oxidation/reduction reactions shifted obviously from the 1^st^ to 5000^th^ cycle, which further verifies a gradual change in the chemical composition of NiCo_2_S_4_. In the aqueous solution of KOH, OH^−^ ions and can serve as sources for the ion-exchange reaction to convert NiCo_2_S_4_ to (Ni, Co) hydroxide.

The redox reactions occurred in both surface and inside of NiCo_2_S_4_ nanosheets and nanowires, which may also result in the structural pulverization and detachment of nanosheet shell from nanowire core. The conversion of chemical composition from NiCo_2_S_4_ to oxide and deterioration of the core-shell structure caused the increase of the resistance of hierarchical NiCo_2_S_4_ CSNAs electrode after 5000 cycles as compared with that before cycling (as evidenced by EIS measurements in Figure S15). In addition, the specific surface area of hierarchical NiCo_2_S_4_ core-shell nanowires was also reduced to 38.9 m^2^/g after 5000 cycles (Figure S16), which is distinctly lower than that before charge/discharge processes (84.9 m^2^/g). As a result of the gradual conversion of NiCo_2_S_4_ to oxide with increased resistivity, the breakdown of the core-shell structure, and the reduced surface area of the NiCo_2_S_4_ CSNAs electrode, the electrochemical performance of the NiCo_2_S_4_ CSNAs electrode, including specific capacity, rate capability and cycling stability, deteriorated after charge/discharge cycles.

As the NiCo_2_S_4_ CSNA electrodes were coated with a surface carbon layer, the NiCo_2_S_4_@C CSNAs still showed their hierarchical morphology after 5000 charge/discharge cycles (Fig. 5d). Interestingly, the TEM observation in Fig. 5e further revealed that the size of shell NiCo_2_S_4_ nanosheets preserved and the NiCo_2_S_4_ core-shell nanowires maintained their integrity without gaps formed at the core-shell interface. In a sharp contrast to the NiCo_2_S_4_ CSNA electrodes, the EDS spectra for the NiCo_2_S_4_@C CSNAs before (i) and after (ii) 5000 cycles in Fig. 5e indicated that the electrode had its chemical composition almost unchanged with only a slight increase of oxygen content after long-term cycling (Figure S17). Correspondingly, the two strong peaks for the faradic oxidation/reduction reactions the CV curves showed only slight shift from 1^st^ to 5000^th^ CV curves (as denoted in Figure S18), which also verifies an unchanged composition of NiCo_2_S_4_@C CSNAs. Based on these observations, the surface carbon layer was demonstrated to play important roles in slowing down the degradation of the NiCo_2_S_4_@C CSNA electrode. First, it could serve as a physical buffer layer to suppress the structural alternation and counteract the pulverization of hierarchical NiCo_2_S_4_ CSNAs. Second, it could function as a chemical blocking layer to retard the chemical composition conversion of the NiCo_2_S_4_ CSNAs during cycling. Finally, it could also provide extra path for direct charge transport to current collector in addition to the conductive NiCo_2_S_4_ core nanowires (Figure S19), which led to a lower resistivity of the hierarchical NiCo_2_S_4_@C CSNAs electrode than that of the NiCo_2_S_4_ CSNAs electrode (Figure S20). Compared with the NiCo_2_S_4_ CSNAs electrode (Figure S19a), the surface carbon layer in the NiCo_2_S_4_@C CSNAs electrode could also reduce repeated charge transfer through the core-shell interface, which could decrease the local Joule heat produced there and the resultant anisotropic volume expansions (-induced detachment of shell nanosheets from core nanowires) (Figure S19b). As a result, NiCo_2_S_4_@C CSNAs electrodes could have a much improved overall electrochemical performance including an increase of specific capacity by (253 mAh g^−1^ vs. 224 mAh g^−1^ at 3 A g^−1^, and 163 mAh g^−1^ vs. 72 mAh g^−1^ at 50 A g^−1^), the rate capability (64.4% vs. 32.3%) and cycling stability (retention of 93.9% vs. 73.3%).

We have successfully synthesized the hierarchical NiCo_2_S_4_@C CSNAs directly on Ni@G foams using hydrothermal reactions followed by a sulfurization process. The hierarchical NiCo_2_S_4_@C CSNAs electrode exhibits exceptionally high specific capacity (253 mAh g^−1^ at 3 A g^−1^), high rate capability (~64.4% retention from at 3 to 50 A g^−1^), and excellent cycling stability (6.1% capacity loss after 5000 cycles at 50 mVs^−1^). By comparatively studying the degradation of hierarchical NiCo_2_S_4_ CSNAs electrode with and without carbon coatings, we revealed that the morphology pulverization, structural separation at core/shell interface, and irretrievably chemical composition change of NiCo_2_S_4_ CSNAs electrode during charge/discharge cycles are major factors that resulted in reduction of the specific capacity, deterioration of the rate capability and long-term cycling stability of the NiCo_2_S_4_ CSNAs electrodes. The surface carbon coating was demonstrated to be able to improve drastically the electrochemical performance of the NiCo_2_S_4_ CSNAs electrodes, which could serve as a physical buffering layer to suppress the structural breakdown, diminish the chemical composition conversion of the NiCo_2_S_4_ CSNAs, and provide extra path for charge transition in addition to the NiCo_2_S_4_ core nanowires. The understanding of the degradation mechanism of the core-shell nanostructured electrodes and as well the beneficial roles of surface carbon coating will be helpful for the design and fabrication of new electrodes for high-performance electrochemical devices.

## Methods

### Synthesis

All chemicals were purchased from Sigma-Aldrich were used as received without further purification. The hierarchical NiCo_2_S_4_ CSNAs, hierarchical NiCo_2_S_4_@C CSNAs and NiCo_2_O_4_ NAs were synthesized on Ni@G foam using hydrothermal reactions followed by a simple sulfurization treatment. The detailed conditions, e.g, source materials, processing temperature, and time, are provided in Supplementary Information I, II and III. The loading weight of hierarchical NiCo_2_S_4_ CSNAs and hierarchical NiCo_2_S_4_@C CSNAs was about 3.4–3.6 mg cm^−2^.

### Characterization

The as-synthesized products were characterized with a D/max-2550 PC X-ray diffractometer (XRD; Rigaku, Cu-Ka radiation), a scanning electron microscope (SEM; S-4800), a transmission electron microscope (TEM; JEM-2100F) equipped with an energy dispersive X-ray spectrometer (EDX), and an X-ray photoelectron spectrometer (ESCALab MKII) with an excitation source of Mg-K radiation. The surface area and pore size distribution of the products were determined by Brunauer-Emmett-Teller (BET) nitrogen adsorption-desorption and Barrett-Joyner-Halenda (BJH) methods (Micromeritics, ASAP2020). The electrical properties of samples were tested *in situ* by using a TEM-scanning tunneling microscope (TEM-STM) holder (commercialized by Nanofactory Instruments AB, GÖteborg, Sweden), which was arranged within a 200 kV high resolution TEM (JEM-2100F) with beam-blank irradiation of a low illumination.

### Electrochemical measurements

Electrochemical measurements were performed on an Autolab Electrochemical Workstation (PGSTAT302N) using a three electrode electrochemical cell and 1 M KOH as the electrolyte. The Ni@G foam supported electroactive materials (1 cm × 1 cm) were directly used as the working electrode. A saturated calomel electrode (SCE) was used as the reference electrode and a platinum (Pt) sheet was used as the counter electrode. All potentials were referred to the reference electrode. The specific capacity and current density were calculated based on the mass of these electroactive materials.

## Additional Information

**How to cite this article**: Zou, R. *et al*. Electrochemical Energy Storage Application and Degradation Analysis of Carbon-Coated Hierarchical NiCo_2_S_4_ Core-Shell Nanowire Arrays Grown Directly on Graphene/Nickel Foam. *Sci. Rep*. **6**, 20264; doi: 10.1038/srep20264 (2016).

## Supplementary Material

Supplementary Information

## References

[b1] Wang G. P., Zhang L. & Zhang J. J. A Review of Electrode Materials for Electrochemical Supercapacitors. Chem. Soc. Rev. 41, 797–828 (2012). 21779609 10.1039/c1cs15060j

[b2] Augustyn V., Simon P. & Dunn B. Pseudocapacitive Oxide Materials for High-rate Electrochemical Energy Storage. Energy Environ. Sci. 7, 1597–1614 (2014).

[b3] Hu C. C., Chang K. H., Lin M. C. & Wu Y. T. Design and Tailoring of the Nanotubular Arrayed Architecture of Hydrous RuO_2_ for Next Generation Supercapacitors. Nano Lett. 6, 2690–2695 (2006). 17163689 10.1021/nl061576a

[b4] Ma R. H., Bando Y. L., Zhang Q. & Sasaki T. Layered MnO_2_ Nanobelts: Hydrothermal Synthesis and Electrochemical Measurements. Adv. Mater. 16, 918–922 (2004).

[b5] Singh A. K., Sarkar D., Khan G. G. & Mandal K. Hydrogenated NiO Nanoblock Architecture for High Performance Pseudocapacitor. ACS Appl. Mater. Interfaces 6, 4684–4692 (2014). 24601472 10.1021/am404995h

[b6] Zhu Y. G. . Phase Transformation Induced Capacitance Activation for 3D Graphene-CoO Nanorod Pseudocapacitor. Adv. Energy Mater. 4, 1301788 (2014).

[b7] Peng S. J. . MS_2_ (M=Co and Ni) Hollow Spheres with Tunable Interiors for High-Performance Supercapacitors and Photovoltaics. Adv. Funct. Mater. 24, 2155–2162 (2014).

[b8] Xia X. H. . Synthesis of Free-Standing Metal Sulfide Nanoarrays via Anion Exchange Reaction and Their Electrochemical Energy Storage Application. Small 10, 766 (2014). 24809111 10.1002/smll.201302224

[b9] Sun C. C. . Phase-controlled synthesis of α-NiS nanoparticles confined in carbon nanorods for High Performance Supercapacitors. Sci. Rep. 4, 7054 (2014). 25394517 10.1038/srep07054 PMC4231331

[b10] Ratha S. & Rout C. S. Supercapacitor Electrodes Based on Layered Tungsten Disulfide-Reduced Graphene Oxide Hybrids Synthesized by a Facile Hydrothermal Method. ACS Appl. Mater. Interfaces 5, 11427–11433 (2013). 24125029 10.1021/am403663f

[b11] Cao X. H., Yin Z. Y. & Zhang H. Three-Dimensional Graphene Materials: Preparation, Structures and Application in Supercapacitors. Energy Environ. Sci. 7, 1850–1865 (2014).

[b12] Rui X. H., Tan H. T. & Yan Q. Y. Nanostructured Metal Sulfides for Energy Storage, Nanoscale 6, 9889–9924 (2014). 25073046 10.1039/c4nr03057e

[b13] Zou R. J. . Three-Dimensional Networked NiCo_2_S_4_ Nanosheet Arrays/Carbon Cloth Anodes for High-Performance Lithium-Ion Batteries, NPG Asia Materials 7, e195 (2015).

[b14] Chen H. C. . Highly Conductive NiCo_2_S_4_ Urchin-Like Nanostructures for High-Rate Pseudocapacitors. Nanoscale 5, 8879–8883 (2013). 23903234 10.1039/c3nr02958a

[b15] Hu L. B. . Highly Conductive Paper for Energy-Storage Devices. P. Natl. Acad. Sci. USA 106, 21490–21494 (2009). 10.1073/pnas.0908858106 PMC2799859 19995965

[b16] Frackowiak E. & Béguin F. Carbon Materials for the Electrochemical Storage of Energy in Capacitors. Carbon 39, 937–950 (2001).

[b17] Liu Q., Jin J. T. & Zhang J. Y. NiCo_2_S_4_@graphene as a Bifunctional Electrocatalyst for Oxygen Reduction and Evolution Reactions. ACS Appl. Mater. Interfaces 5, 5002–5008 (2013). 23662625 10.1021/am4007897

[b18] Du W. M. . One-Step Synthesis of CoNi_2_S_4_ Nanoparticles for Supercapacitor Electrodes. RSC Adv. 4, 6998–7002 (2014).

[b19] Shen L. F. . NiCo_2_S_4_ Nanosheets Grown on Nitrogen-Doped Carbon Foams as an Advanced Electrode for Supercapacitors. Adv. Energy Mater. 5, 1400977 (2015).

[b20] Yang J. Q. . Hierarchical porous NiCo_2_S_4_ hexagonal plates: Formation via chemical conversion and application in high performance supercapacitors. Electrochimica Acta 144, 16–21 (2014).

[b21] Wan H. Z. . NiCo_2_S_4_ Porous Nanotubes Synthesis via Sacrificial Templates: High-Performance Electrode Materials of Supercapacitors. CrystEngComm 15, 7649–7651 (2013).

[b22] Zhang Z. Y. . NiCo_2_S_4_ Sub-Micron Spheres: An Efficient Non-Precious Metal Bifunctional Electrocatalyst. Nanoscale 6, 3540–3544 (2014). 24595310 10.1039/c3nr05885a

[b23] Cong S., Tian Y. Y., Li Q. W., Zhao Z. G. & Geng F. X. Single-Crystalline Tungsten Oxide Quantum Dots for Fast Pseudocapacitor and Electrochromic Applications. Adv. Mater. 26, 4260–4267 (2014). 24740967 10.1002/adma.201400447

[b24] Oh P. . Superior Long-Term Energy Retention and Volumetric Energy Density for Li-rich Cathode Materials. Nano Lett. 14, 5965–5972 (2014). 25180657 10.1021/nl502980k

[b25] Xiao J. W., Wan L., Yang S. H., Xiao F. & Wang S. A. Design Hierarchical Electrodes with Highly Conductive NiCo_2_S_4_ Nanotube Arrays Grown on Carbon Fiber Paper for High-Performance Pseudocapacitors. Nano Lett. 14, 831–838 (2014). 24437988 10.1021/nl404199v

[b26] Chen H. C. . *In Situ* Growth of NiCo_2_S_4_ Nanotube Arrays on Ni Foam for Supercapacitors: Maximizing Utilization Efficiency at High Mass Loading to Achieve Ultrahigh Areal Pseudocapacitance. J. Power Sources 254, 249–257 (2014).

[b27] Tian W. . & Ni(OH)2 Nanosheet@Fe_2_O_3_ Nanowire Hybrid Composite Arrays for High-Performance Supercapacitor Electrodes. Nano Energy 2, 754–763 (2013).

[b28] Wang X. . Cu/Li_4_Ti_5_O_12_ Scaffolds as Superior Anodes for Lithium-Ion Batteries. NPG Asia Materials 7, e171 (2015).

[b29] Guan H., Wang X., Chen S. M., Bando Y. & Golberg D. Coaxial Cu-Si@C Array Electrodes for High-Performance Lithium Ion Batteries. Chem. Commun. 47, 12098–12100 (2011). 10.1039/c1cc15595d 22006078

[b30] Kung C. W. . CoS Acicular Nanorod Arrays for the Counter Electrode of an Efficient Dye-Sensitized Solar Cell. ACS Nano 6, 7016–7025 (2012). 22747428 10.1021/nn302063s

[b31] Zhang Y. F. . Shape-Controlled Synthesis of NiCo_2_S_4_ and Their Charge Storage Characteristics in Supercapacitors, Nanoscale 6, 9824–9830 (2014). 25027699 10.1039/c4nr02833c

[b32] Peng T. . Construction of Mass-Controllable Mesoporous NiCo_2_S_4_ Electrodes for High Performance Supercapacitors. J. Mater. Chem. A 2, 19376–19382 (2014).

[b33] Liu Q., Zou R. J., Bando Y., Golberg D. & Hu J. Q. Nanowires Sheathed Inside Nanotubes: Manipulation, Properties and Applications. Prog. Mater. Sci. 70, 1–49 (2015).

[b34] Zou R. J. . Melting of Metallic Electrodes and Their Flowing Through a Carbon Nanotube Channel within a Device. Adv. Mater. 25, 2693–2699 (2013). 23559074 10.1002/adma.201300257

[b35] Zou R. J. . High Detectivity Solar-Blind High-Temperature Deep-Ultraviolet Photodetector Based on Multi-Layered (l00) Facet-Oriented β-Ga_2_O_3_ Nanobelts. Small 10, 1848–1856 (2014). 24520013 10.1002/smll.201302705

[b36] Zou R. J., Yu L., Zhang Z. Y., Chen Z. G. & Hu J. Q. High-Precision, Large-Domain Three-Dimensional Manipulation of Nano-Materials for Fabrication Nanodevices. Nanoscale Res. Lett. 6, 473 (2011). 21794151 10.1186/1556-276X-6-473 PMC3211986

[b37] Simon P., Gogotsi Y. & Dunn B. Where Do Batteries End and Supercapacitors Begin? Science 343, 1210–1211 (2014). 24626920 10.1126/science.1249625

[b38] Conway B. E. Transition from “Supercapacitor” to “Battery” Behavior in Electrochemical Energy Storage. J. Electrochem. Soc. 138, 1539–1548 (1991).

[b39] Lindström H. . Li^+^ Ion Insertion in TiO_2_ (Anatase). 2. Voltammetry on Nanoporous Films. J. Phys. Chem. B 101, 7717–7722 (1997).

[b40] Xia X. H. . Synthesis of Free-Standing Metal Sulfide Nanoarrays via Anion Exchange Reaction and Their Electrochemical Energy Storage Application. Small 10, 766–773 (2014). 24809111 10.1002/smll.201302224

[b41] Zhou W. J. . One-step synthesis of Ni_3_S_2_ nanorod@Ni(OH)_2_ nanosheet core-shell nanostructures on a three-dimensional graphene network for high-performance supercapacitors. Energy Environ. Sci. 6, 2216–2221 (2013).

[b42] Guan C. . Hollow Core-Shell Nanostructure Supercapacitor Electrodes: Gap Matters. Energy Environ. Sci. 5, 9085–9090 (2012).

[b43] Xia X. H. . High-Quality Metal Oxide Core/Shell Nanowire Arrays on Conductive Substrates for Electrochemical Energy Storage. ACS Nano 6, 5531–5538 (2012). 22545560 10.1021/nn301454q

[b44] Sun J. Q. . 3D Core/Shell Hierarchies of MnOOH Ultrathin Nanosheets Grown on NiO Nanosheet Arrays for High-Performance Supercapacitors. Nano Energy 4, 56–64 (2014).

[b45] Zou R. J. . Dendritic Heterojunction Nanowire Arrays for High-Performance Supercapacitors. Sci. Rep. 5, 7862 (2015). 25597402 10.1038/srep07862 PMC4297956

