# Supplementary material for: Electrochemical Energy Storage Application and Degradation Analysis of Carbon-Coated Hierarchical NiCo2S4 Core-Shell Nanowire Arrays Grown Directly on Graphene/Nickel Foam
Source: Sci Rep. 2016 Feb 1;6:20264. doi: 10.1038/srep20264 (PMC4735299; doi:10.1038/srep20264)
Supplement: Supplementary Information [file srep20264-s1.doc]

**Supporting Information**

Electrochemical Energy Storage Application and Degradation Analysis of Carbon-Coated Hierarchical NiCo_2_S_4_ Core-Shell Nanowire Arrays Grown Directly on Graphene/Nickel Foam

Rujia Zou^1,2^, Muk Fung Yuen^2^, Li Yu^1^, Junqing Hu^1^,*, Chun-Sing Lee^2^ & Wenjun Zhang^2^,*

^1^State Key Laboratory for Modification of Chemical Fibers and Polymer Materials, College of Materials Science and Engineering, Donghua University, Shanghai 201620, China and ^2^Center of Super-Diamond and Advanced Films (COSDAF), Department of Physics and Materials Science, City University of Hong Kong, Hong Kong


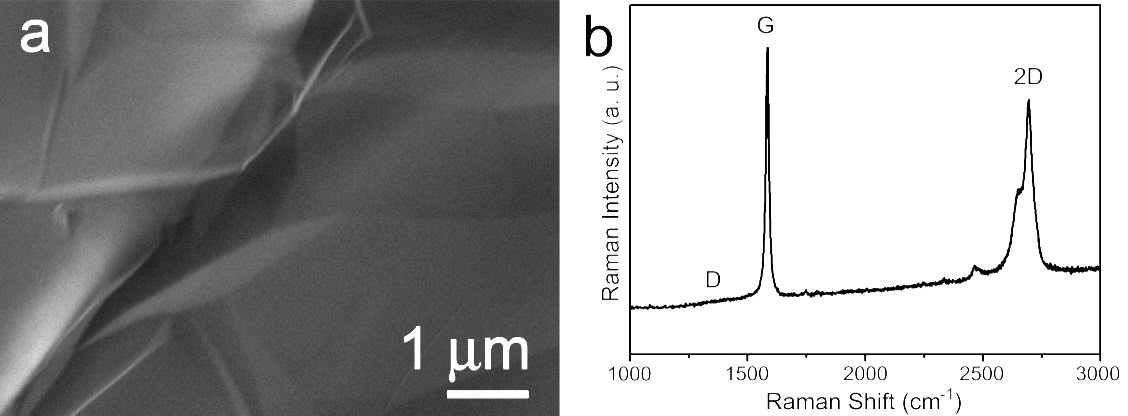


**Figure S1.** **(a)** SEM image of Ni@G foam. **(b)** Raman spectra of graphene on Ni foam.


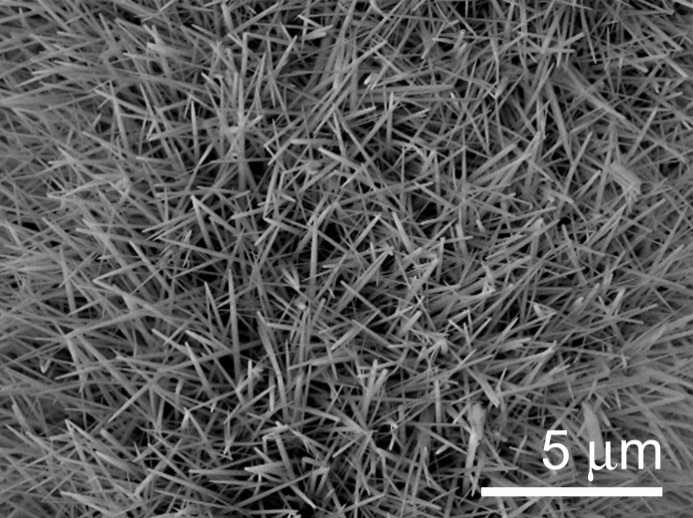


**Figure S2.** SEM image of (Ni, Co) hydroxide nanowire arrays growth on Ni@G foam.


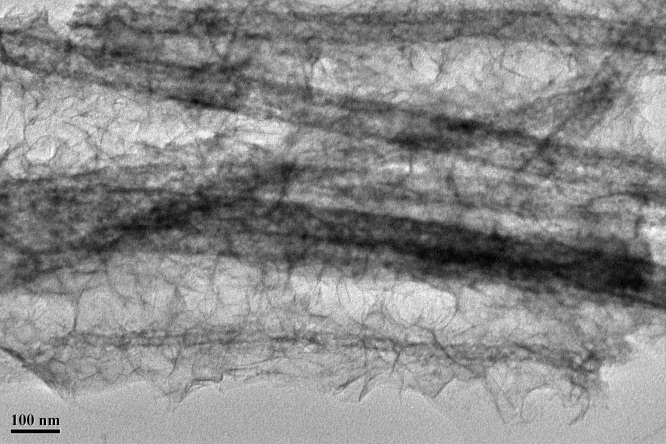


**Figure S3.** TEM image of the hierarchical NiCo_2_S_4_ core-shell nanowires.


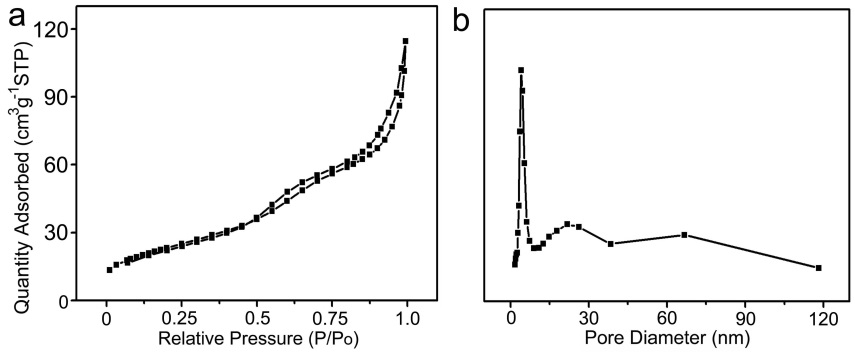


**Figure S4.** **(a)** N_2_ adsorption/desorption isotherms and **(b)** the corresponding pore size distribution of the hierarchical NiCo_2_S_4_ core-shell nanowires scratched from Ni@G foam.


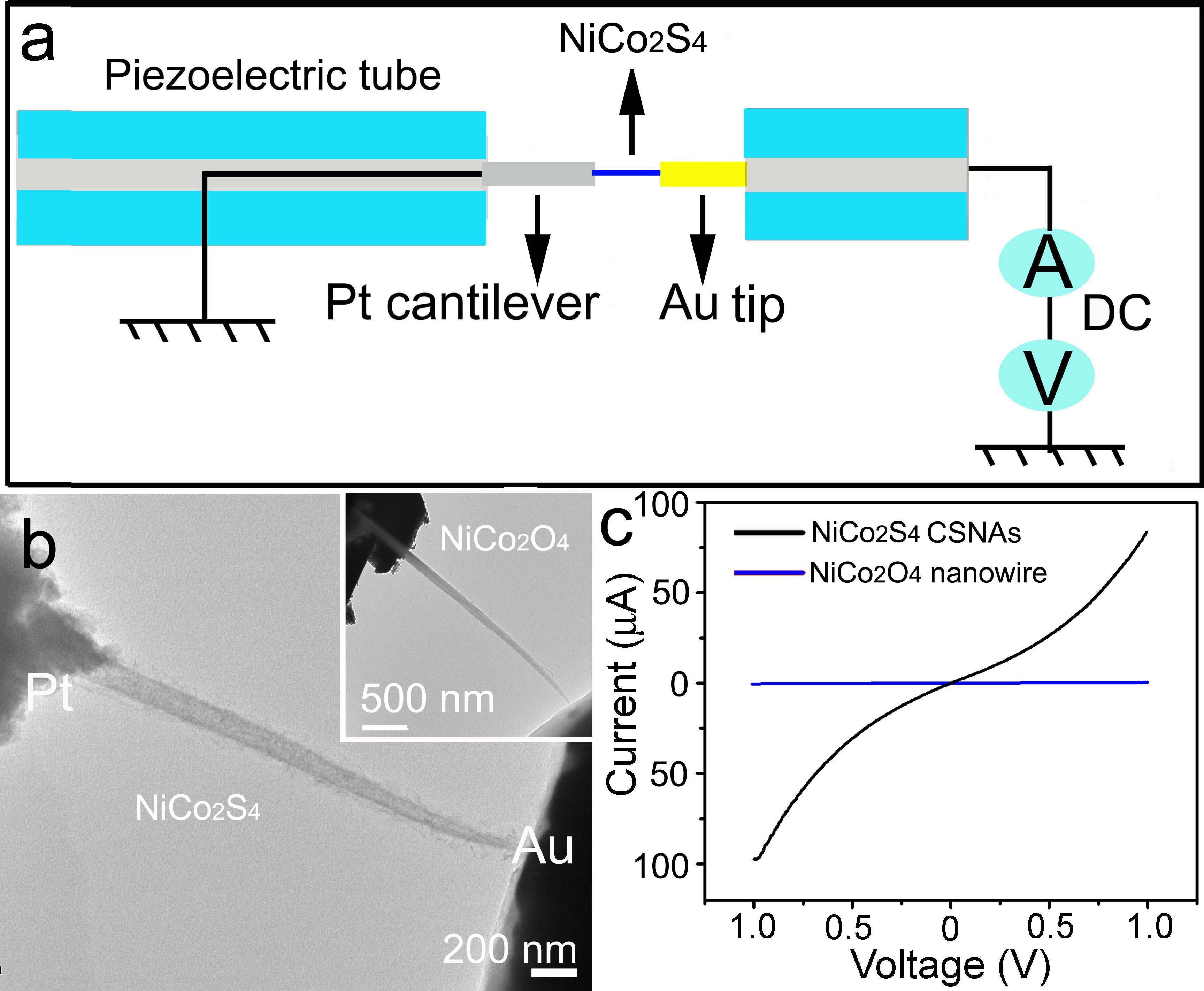


**Figure S5.** **(a)** The experimental setup for the electrical property test of individual nanowire. **(b)** TEM image of the hierarchical NiCo_2_S_4_ core-shell nanowire between the cantilever and tip; inset shown TEM image of the NiCo_2_O_4_ nanowire between the cantilever and tip. **(c)** The corresponding I-V curves for the NiCo_2_S_4_ nanowire (black curve) and NiCo_2_O_4_ nanowire (blue curve).


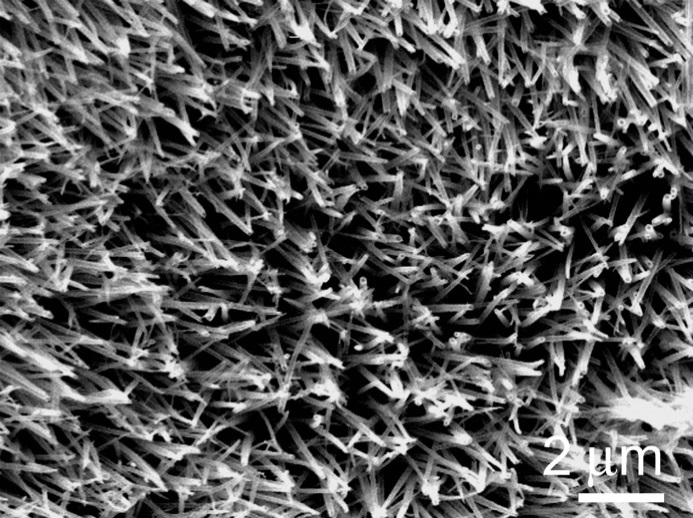


**Figure S6.** SEM image of NiCo_2_S_4_ nanowire arrays growth on Ni@G foam.


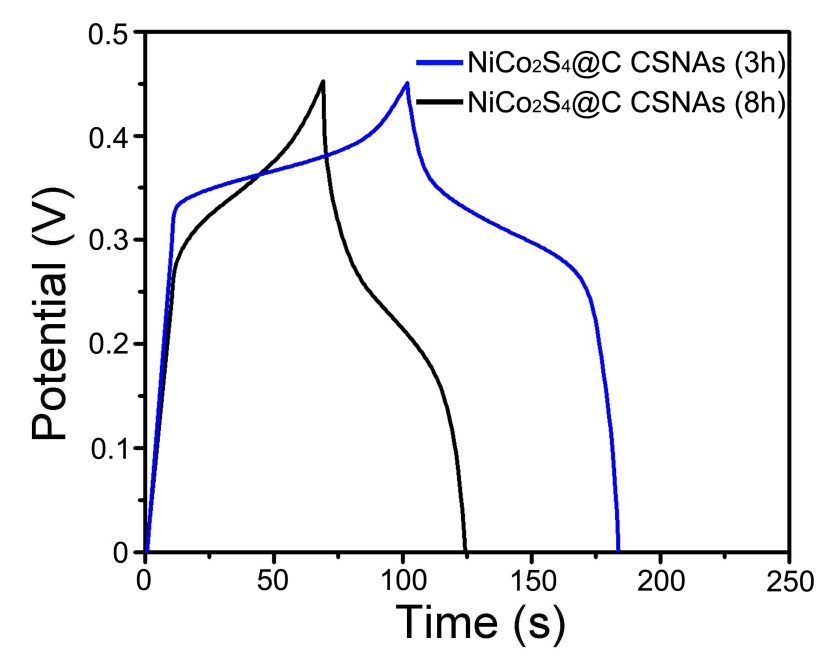


**Figure S7.** Galvanostatic CD curves of NiCo_2_S_4_@C CSNAs with carbon layer synthesized by hydrothermal reaction for 3h and 8h.The current density was maintained the same at 10 A g^-1^ for the test.


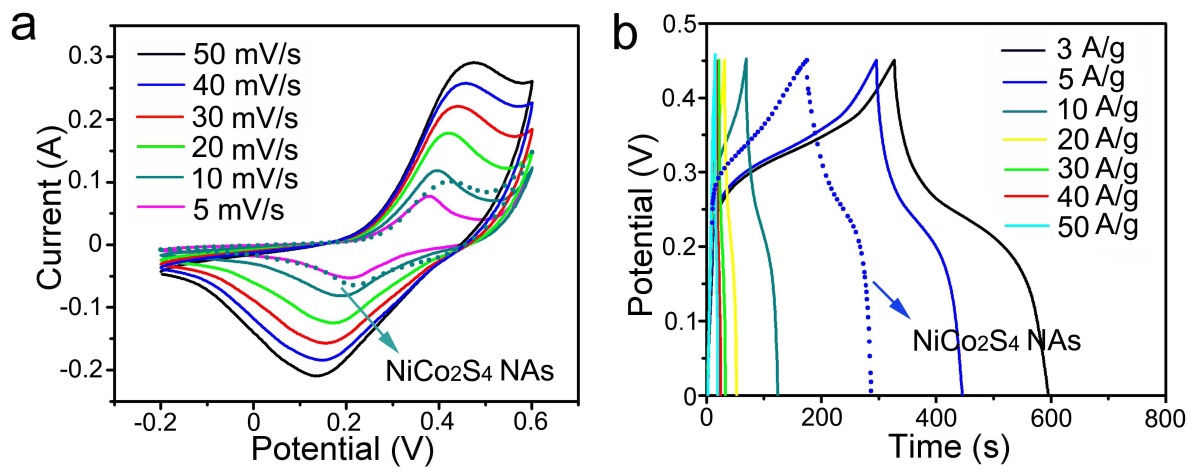


**Figure S8.** Electrochemical properties of hierarchical NiCo_2_S_4_ CSNAs and NiCo_2_S_4_ NAs electrodes in 1 M KOH aqueous solution at room temperature. **(a)** The CVcurves of the NiCo_2_S_4_ CSNAs electrode at different scan rates. The CV curve of NiCo_2_S_4_NAs electrode measured at a scan rate of 10 mV/s is also shown by the green dotted line as a reference. **(b)** The CD curves of the NiCo_2_S_4_ CSNAs electrode at different current densities. The CD curve of NiCo_2_S_4_NAs electrode measured at a scan rate of 10 A g^-1^ is also plotted (the blue dotted line) as a reference.


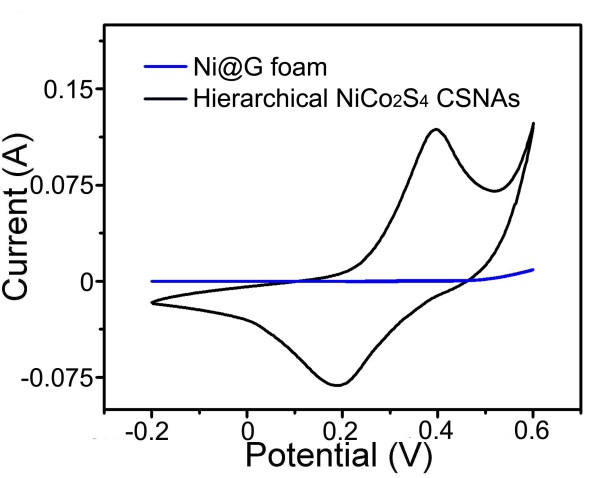


**Figure S9.** The CV curves of the NiCo_2_S_4_ NAs electrode and Ni@G foam measured at a scan rate of 10 mV/s.


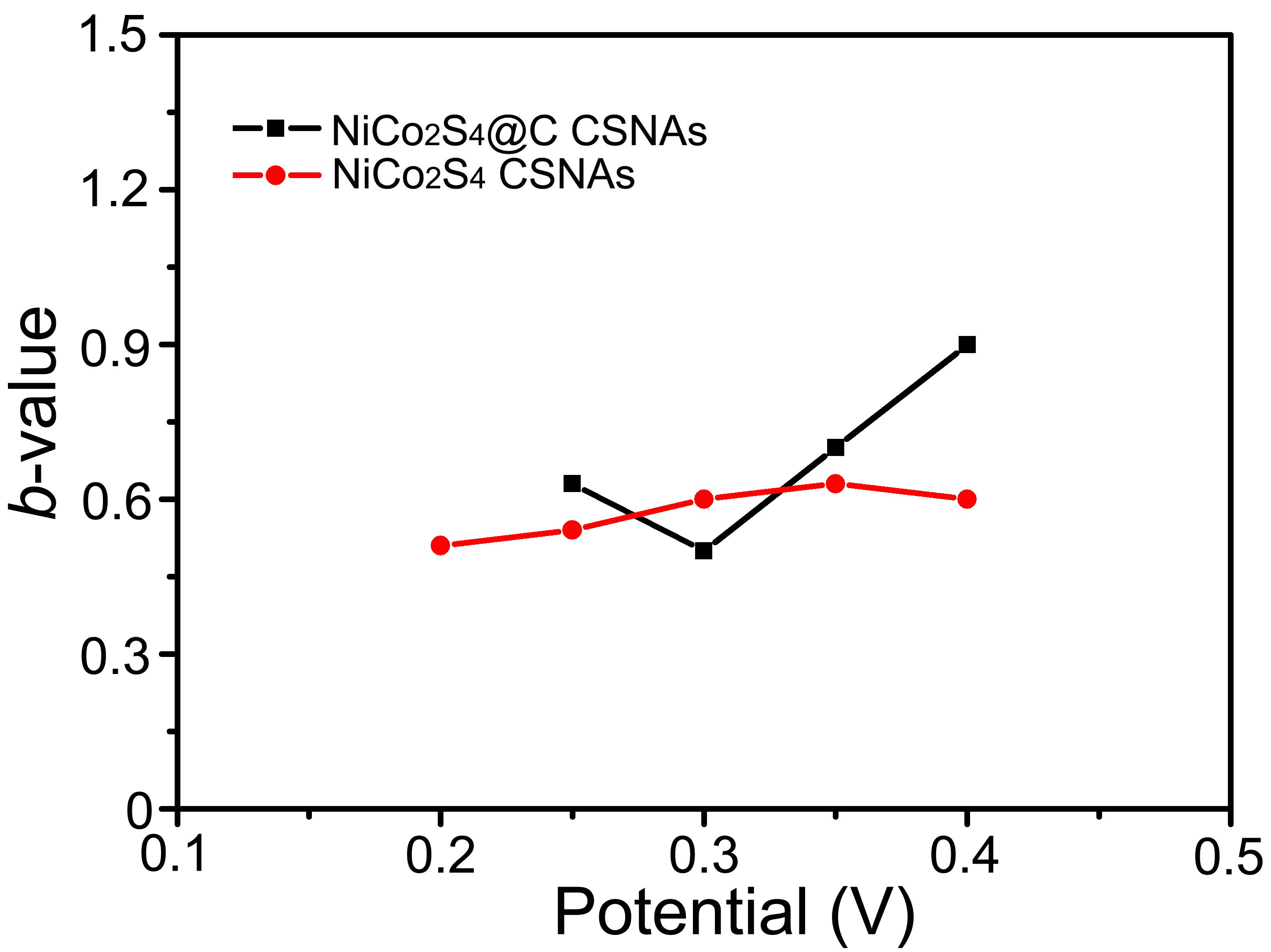


**Figure S10**. *b-*values for hierarchical NiCo_2_S_4_@C CSNAs and NiCo_2_S_4_ CSNAs electrodes upon potentials for cathodic sweeps. b-values were obtained by from power-law relationships of the current (*i*) with the sweep rate (*v*) at a fixed potential, i.e., *i=av^b^*, where *a* and *b* are adjustable parameters.^1-3^ Over a wide range of sweep rates *v*, whereas a *b*-value of 0.5 would indicate that the current is controlled by semi-infinite linear diffusion (battery materials), a value of 1 indicates that the current is surface-controlled (pseudocapacitor materials).^3^ The calculated *b-*values for hierarchical NiCo_2_S_4_@C CSNAs and hierarchical NiCo_2_S_4_ CSNAs electrodes at the potential window range from 0.2 to 0.4 V in reduction processes indicate they are closer to the battery-type materials.


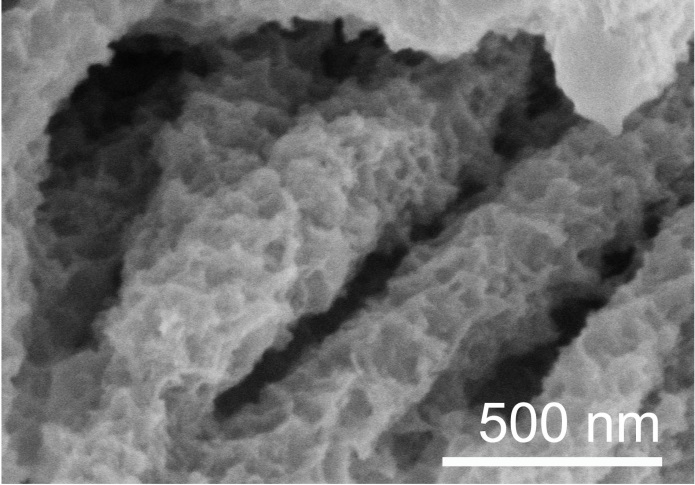


**Figure S11.** Enlarged SEM image of NiCo_2_S_4_ CSNAs electrodes after charge/discharge for 5000 cycles at a scan rate of 50 mV s^-1^.


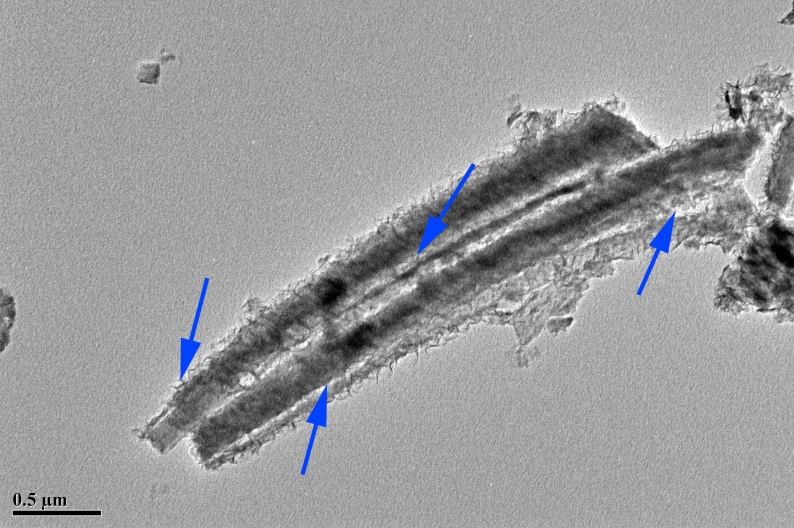


**Figure S12.** TEM images of NiCo_2_S_4_core-shell nanowires after charge/discharge for 5000 cycles at the scan rate of 50 mV s^-1^.


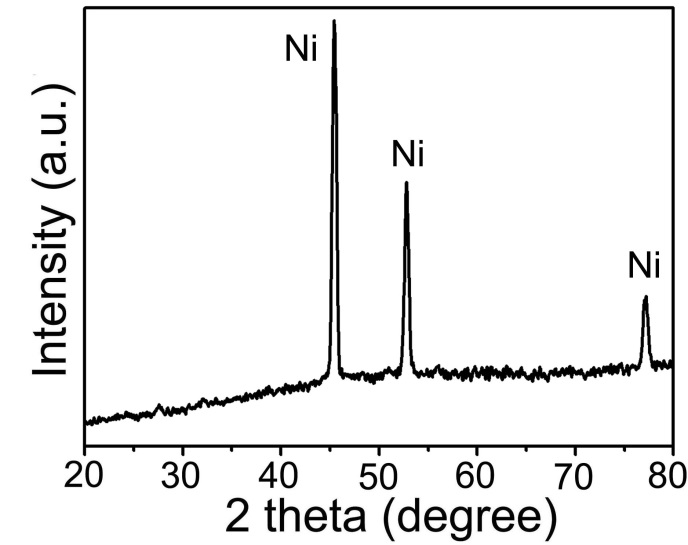


**Figure S13.** XRD spectrum of hierarchical NiCo_2_S_4_ CSNAs on the Ni@G foam after discharging/charging for 5000 cycles at the scan rate of 50 mV s^-1^.


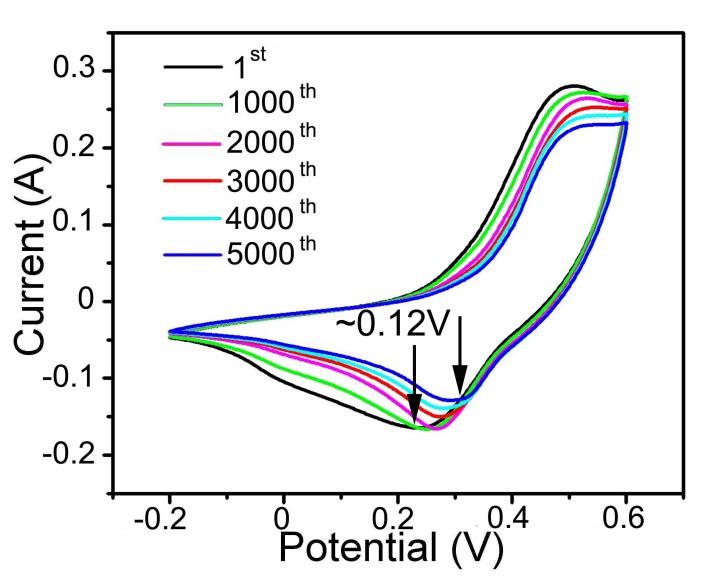


**Figure S14.** CV curves of hierarchical NiCo_2_S_4_ CSNAs at a scan rate of 50 mV s^-1^ for the 1^st^, 1000^th^, 2000^th^, 3000^th^, 4000^th^ and 5000^th^ cycle.


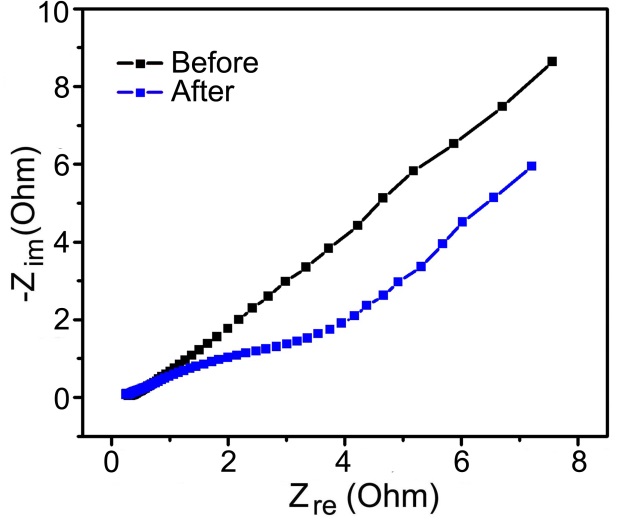


**Figure S15.** Electrochemical impedance spectra before and after 5000 cycles.


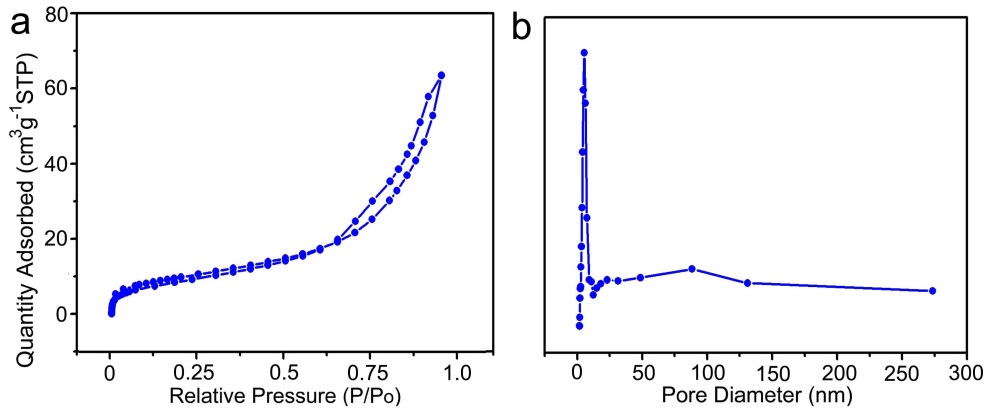


**Figure S16.** **(a)** N_2_ adsorption/desorption isotherms and **(b)** the corresponding pore size distribution of the hierarchical NiCo_2_S_4_ core-shell nanowire scratched from Ni@G foam after 5000 cycles.


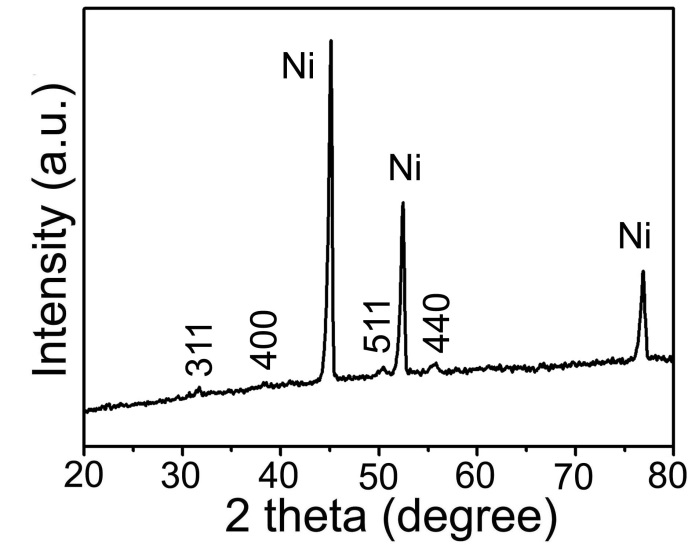


**Figure S17**. XRD spectrum of hierarchical NiCo_2_S_4_@C CSNAs on the Ni@G foam after discharging/charging for 5000 cycles at the scan rate of 50 mV s^-1^.


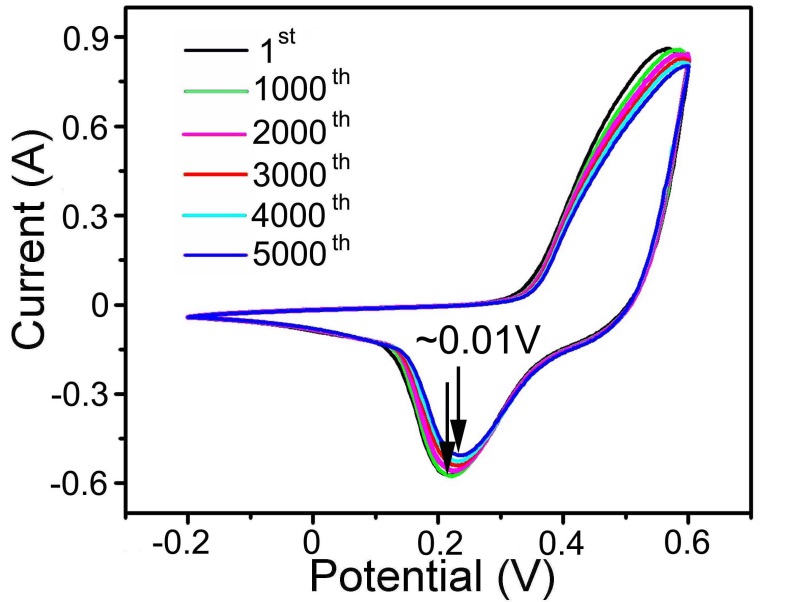


**Figure S18.** CV curves of hierarchical NiCo_2_S_4_@C CSNAs electrode at a scan rate of 50 mV s^-1^ for the 1^st^, 1000^th^, 2000^th^, 3000^th^, 4000^th^ and 5000^th^ cycle.


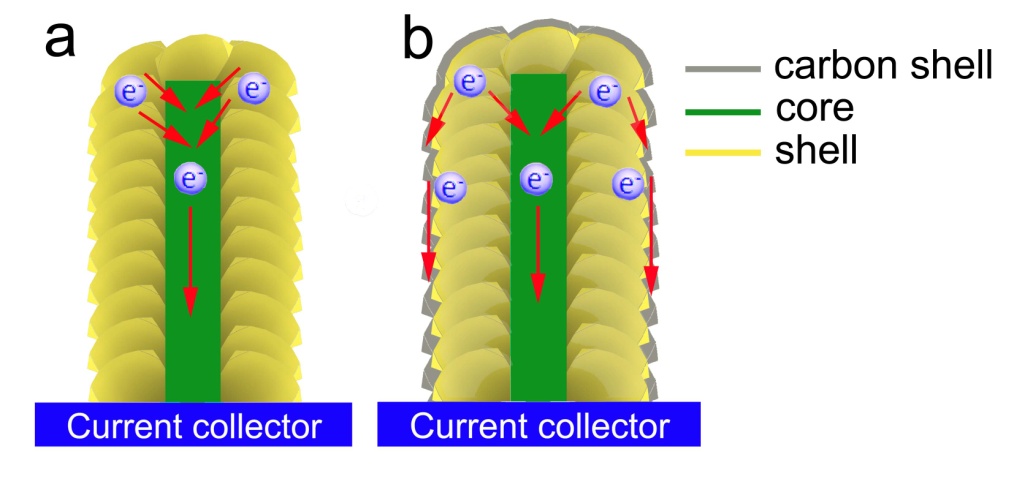


**Figure S19**. Schematic representation of electron paths on the hierarchical NiCo_2_S_4_ CSNAs electrode and hierarchical NiCo_2_S_4_@C CSNAs electrode.


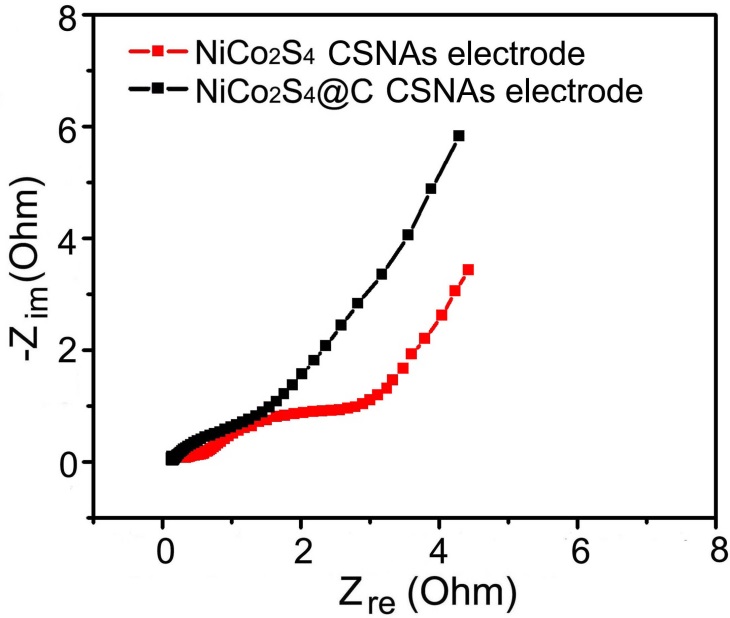


**Figure S20.** EIS of the hierarchical NiCo_2_S_4_@C CSNAs (black curve) and hierarchical NiCo_2_S_4_ CSNAs (red curve) electrodes.

**I Growth of hierarchical NiCo_2_S_4_ CSNAs and NiCo_2_S_4_@C CSNAs on Ni@G foam.**

Graphene was first deposited on Ni foamby chemical vapor deposition (CVD). The Ni foams were put in a horizontal tube furnace and heated to 1000 ºC under the gas flow of Ar (200 sccm) and H_2_ (20 sccm) for 15 min to clean the surfaces of Ni foams and reduce the oxide layer. Then, the temperature was reduced to 950 °C, and ethanol was bubbled into the reaction tube by Ar (200 sccm) and H_2_ (40 sccm) flow under an ambient pressure. After reaction for 15 min, the tube was rapidly cooled down to room temperature at a rate of about 100 ºC min^-1^ under the protection of Ar (280 sccm) and H_2_ (20 sccm).

To synthesize NiCo_2_S_4_CSNA, NiCo_2_O_4_-precursor nanowire array was first grown on Ni@Gfoam by hydrothermal method. In this step, 1.185g CoCl_2_·6H_2_O, 0.657g NiCl_2_·6H_2_O and 0.45g urea were dissolved into a solution of 40 mL of methanol to form a clear pink solution in a 50 mL autoclave. After being cleaned by sonication in ethanol for 30 min, the Ni@G foam was transferred into the autoclave and kept in an electric oven at 140 ˚C for 8 h. The Ni@G foam was taken out from autoclave at room temperature and cleaned to remove the loosely attached products on its surface (It should be noted that, in the case by applying ultrasonic cleaning here, NiCo_2_S_4_NA without the hierarchical core-shell configuration was obtained, which was used as a control sample in this work). Then the 3D hierarchical NiCo_2_S_4_CSNA was obtained by sulfurization, i.e., immergingthe Ni@Gwith NiCo_2_O_4_-precursor nanowires in a solution containing Na_2_S·xH_2_O (0.60 g) in an autoclave and keeping at 100 ˚C for 48 hin an electric oven. The as-obtained product was washed successively with deionized water and ethanol to remove any residual ionic species, and dried in vacuum for 24 h.

For the carbon coating on the hierarchical NiCo_2_S_4_ CSNA, the sample transferred into autoclave with anaqueous solution of glucose (0.15 M) and maintained at 180 ˚C for 3 h in an electric oven. After cooling to room temperature naturally, the sample was collected and washed with deionized again. Finally, both samples with and without carbon coatings (NiCo_2_S_4_@C CSNA and references of NiCo_2_S_4_ NA and NiCo_2_S_4_CSNA) were annealed in Ar gas at 320 °C for 2 h with a heating rate of 0.5 °C min.

**II Growth of NiCo_2_O_4_ nanowires.**

1.185g CoCl_2_·6H_2_O, 0.657g NiCl_2_·6H_2_O, and 0.45g urea were dissolved into a 40 mL methanol to form a clear pink solution in a 50 mL autoclave. The Ni foam was cleaned by sonication in ethanol for 30 min, and then transferred into the autoclave and kept at 140 ˚C for 8 h in an electric oven. The Ni foam with NiCo_2_O_4_-precursor nanowire array was taken out from autoclave at room temperature and then cleaned by ultrasonication to remove the attached products. The sample was annealed in air at 320 °C for 2 h with a heating rate of 0.5 °C min to improve the crystallinity of NiCo_2_O_4_ nanowires.

**Reference:**

1 Simon, P., Gogotsi, Y. & Dunn, B. Where Do Batteries End and Supercapacitors Begin? *Science* **343**, 1210-1211 (2014).

2 Conway, B. E. Transition from "Supercapacitor" to "Battery" Behavior in Electrochemical Energy Storage. *J. Electrochem. Soc.* **138**, 1539-1548 (1991).

3 Lindström, H., So1dergren, S., Solbrand, A., Rensmo, H., Hjelm, J., Hagfeldt, A. & Lindquist, S. Li^+^ Ion Insertion in TiO_2_ (Anatase). 2. Voltammetry on Nanoporous Films. *J. Phys. Chem. B* **101**, 7717-7722 (1997).
